# General Medicine and Therapeutics

**Published:** 1896-06

**Authors:** 


### GENERAL MEDICINE AND THERAPEUTICS.

EDITED BY DR. LUTHER B. GRANDY.

### ACUTE BRONCHITIS IN CHILDREN.

The following prescriptions and suggestions have been collected by *Pediatrics* (April):

It is usually wise to begin the treatment with a laxative of castor oil or calomel. During the first twelve or twenty-four hours an emetic in conjunction with a warm bath may do good.

℞ Powdered ipecac ........................................ gr. i to gr. ii.
Syrup of ipecac ........................................ ℥ i.

Mix. Sig. A teaspoonful every ten minutes until the desired effect is produced.—*Perrier.*

Counter-irritation over the chest is usually indicated, with a thin mustard plaster, camphorated oil, oil and turpentine, or the following:

℞ Oil of cloves ........................................ ʒ ii.

Camphorated oil ........................................ ℥ iv.

Mix. Sig. For external use.—*J. Lewis Smith.*

Warm drinks should be given, particularly hot milk, and, if the fever is high, one or two grains of quinine may be given in a little coffee or aromatic syrup of yerba santa. The air of the room must be kept fresh, and moderately warm and moist. The vapor of turpentine is often beneficial.

Flaxseed poultices covered with oil-silk are useful, especially for the relief of pain, and cold compresses are useful not only to reduce the fever, but as a stimulant, which causes deep inspirations.

℞ Mist. Glycyrrh. Co ........................................ ℥ iv.

Sig. Half a teaspoonful every two (2) hours to a child of three years.

℞ Syr. ipecac,

Spts. ether. nitros., aa ........................................ ʒ ii.

Ol. ricini ........................................ ʒ iii.

Syr. bal. tolut ........................................ ℥ i.

Misce. Dose: Half a teaspoonful to one teaspoonful, every second hour, for the age of one to two years.—*J. Lewis Smith.*

℞ Ammonii chloridi ........................................ ʒ i.

Syr. bal. tolut ........................................ ℥ ii.

Mix. Dose: At the age of three months, fifteen drops; at six months, thirty drops, in water.

℞ Vini ipecacuanhæ ........ *m* v.
Liq. ammonii acetatis........ *m* x.
Glycerini ........ *m* xv.
Aquam floræ aurantii........ *ad* ʒ i.
Misce. F. haustus.
Sig. For an infant, to be taken every four hours.—*Eustace Smith.*

An opiate affords marked relief from the annoyance of the cough, especially at night.

℞ Tinct. opii camph........ ʒ i-iv.

Syrup tolutani........ ℥ i.

Aquæ........ *ad* ʒ iiss.

Mix. Sig. Shake and take one teaspoonful.—*F. Gordon Morrill.*

℞ Liq. opii composit........ gtt. x.
Potas. bromidi........ ʒ i.
Syr. rubi idæi (raspberry)........ ℥ i.
Aquæ........ ℥ iss.
Misce. Sig. One teaspoonful when needed for restlessness and loss of sleep from cough.—*J. Lewis Smith.*

When the mucous flow has become well established, one of the following mixtures may be prescribed:

℞ Tinct. scillæ........ *m* xx.
Syrup tolutani,
Syrup pruni Virgin., aa........ ℥ ss.
Aquæ........ *ad* ℥ ii ss.
Misce. Sig. Shake and take one teaspoonful for a child one year old.—*F. Gordon Morrill.*

℞ Ammonii carb........ gr. v-x.
Tinct. scillæ........ *m* xxv.
Syrup senegæ........ ℥ ss.
Syrup pruni Virgin........ ℥ iii.
Misce. Sig. Shake and take one teaspoonful three or four times a day, for a child one year old.—*F. Gordon Morrill.*

As the disease progresses, if bronchial secretion becomes very profuse, one of the following prescriptions may be used:

℞ Oxide of antimony........ gr. ss.
Syrup of senega........ ʒ i.
Syrup of acacia........ ℥ ii.
Mix. Sig. A teaspoonful every two hours.—*Perrier.*

℞ Terpine hydrate........ gr. ii-iv.
Brandy........ ʒ ii.
Syrup of cinchona........ ʒ ss.
Syrup of orange........ ℥ ii.
Mix. Sig. A teaspoonful every two hours.—*Perrier.*

The coal-tar antipyretics often have a marked effect in diminishing the amount of secretion, lessening the severity and frequency of the cough and relieving the pain.

℞ Phenacetin........................................................ gr. xii-xxiv.
Caffein.................................................................. gr. i-ii.
Mix. Div. in chart. No. xii (12).
Sig. Give one powder every four (4) hours.—*W. S. Christopher.*

℞ Phenocoll............................................................ gr. xv-xxx.
Camph. monobromat................................................ gr. ii.
Caffein citratis...................................................... gr. ii.
Mix. Div. in chart No. xii (12).
Sig. One powder every four (4) hours.—*Dillon Brown.*

Again, it must be remembered that certain cases of bronchitis may be of indirect origin, depending upon some affection of the heart, of the stomach, of the liver, and even of the kidneys; of the skin, as in erysipelas; or it may be due to typhoid fever, or measles, or to the elimination of certain medicinal substances; and, finally, it may be of diathetic origin, as gout or rheumatism.

℞ Tinct digitalis ..................................................... *m* xx.

Tinct. conii............................................................ ʒ i.

Pulv. ipecac .......................................................... gr. i-ii.

Oxymel. scillæ....................................................... ℥ i.

Muc. acaciæ .......................................................... ʒ iiiss.

Misce. Sig. A teaspoonful every three hours when the bronchitis is associated with disease of the heart.—*Ferrand.*

℞ Tinct. nucis vomicæ,
Tinct. digitalis, aa................................................. ʒ ss-i.
Tinct. cardamomi comp........................................... *ad* ℥ ii.
Mix. Sig. A teaspoonful in water four (4) times a day in the cases following upon influenza.—*Thomas Grainger Stewart.*

℞ Sodii salicylatis,
Potassii bicarbonatis, aa......................................... ʒ i.
Syrupi tolutani....................................................... ℥ ss.
Aquæ anisi ............................................................ *ad* ℥ vi.
Mix. Sig. A tablespoonful in water four (4) times a day in the cases depending upon a rheumatic diathesis.

℞ Vini colchici.......................................................... ʒ i.
Vini antimonialis.................................................... ʒ ss.
Potassii bicarbonatis............................................... ʒ iss.
Aquæ destillatæ ..................................................... *ad.* ℥ vi.
Mix. Sig. A tablespoonful in water four (4) times a day in cases depending upon a gouty diathesis.—*Thomas Grainger Stewart.*

Dry cupping over the back of the chest or a few leeches over the lateral parts of the chest may be extremely beneficial.

### THE PLEA OF INSANITY.

This subject has afforded a text for many able papers from the pens of scientific writers, and called forth animated discussions in medical and medico-legal societies for years. Yet more and more frequent has become the plea of insanity as a defense when a person has committed murder or some other heinous crime. The extent to which this plea has been carried, and the success it has met with, has become a cause for gravest alarm. Hardly a week passes that we do not read in the daily papers of a cold-blooded murder where the statement is made that the attorneys have not, as yet, decided whether to enter a plea of self-defense or insanity. A man commits a murder, disguises himself, tries to escape, is finally captured and brought back to the scene of his crime, and immediately begins to act foolish. His attorney, and often the officers about the jail, gravely announce that he is insane. It is proven to the satisfaction of the jury (honest but ignorant) that he is really insane. He is then sent to an asylum for a few months, and finally discharged as cured, to go forth into the world and murder *ad libitum,* without the fear of anything worse than a few months in an asylum, for he has already been proven insane, which, with the help of so-called experts, is easy to do again and again.

Only a short time ago in this county, a man killed another with a coupling-pin, robbed him of $40 and divided it with an accomplice. He denied all knowledge of it when accused, but "broke down and confessed all" when told that his companion in crime was in jail and had told everything. This cold-blooded assassin was pronounced not guilty on the plea of insanity, and sent to our "poor farm" for safe-keeping. His attorney, in a few weeks thereafter went before the county court and asked to have him released, giving the court as a reason for such action that should he be kept longer among the paupers and insane it would surely drive him crazy. The court released him. Now, if this man was insane, for the protection of society, he should have been confined for life, for it was offered in evidence that he had been "weak-minded and foolish from childhood." Could the commission of one murder, and a few months' confinement at the "poor farm," cure him of a lifetime malady? But it is not necessary to cite cases or to go into detail, for every reader of the public press is familiar with such cases. The remedy is what we want.

The one we shall advocate is not new, but to our mind is certainly the best. When a prisoner pleads insanity as a defense for crime, the court should be empowered to appoint a commission of insanity experts to examine (with plenty of time) into and pass on his case. If they agree that he is insane, let the court order him into confinement, at least, until those who are capable of judging shall pronounce him sane. If, on the other hand, he should be pronounced sane, then try him on the evidence, eliminating the insanity dodge. When this is done the plea of insanity as a defense will rapidly disappear.

This is an old subject, but a subject must be considered new and unsettled until settled right. We have thus briefly called attention to this rapidly growing abuse in the hope that the medical profession, and through it the public, may be induced to "rise in their might" and legally put a stop to a practice which is a menace and a disgrace to our civilization.—*Langsdale's Lancet.*

---

### KEEP AN EYE ON CUBA.

The report reaches us that yellow fever is rapidly developing in Cuba, and it behooves the authorities at Quarantine to be on the alert. In these days it should be a matter of exceeding difficulty for yellow fever to gain a foothold here. Still, in view of the fact that the protracted war in Cuba has necessarily interfered with the proper sanitation which is gradually rooting out epidemics from all civilized neighborhoods, it is peculiarly essential that the health officer of the port should keep in touch with the American representatives at Havana in order that the embarkation at that point of all suspicious cases may be prevented.—*American Medico-Surgical Bulletin.*

---

Doctor—"Your wife is in a critical state; I should advise you to call in some specialist to consult on the case."

Husband—"I told my wife long ago she ought to get proper medical advice, but she thought you would be offended."—*American Druggist.*

